# Hot pants: The emerging field of exercise mimetics, from hospital beds to the international space station

**DOI:** 10.14814/phy2.70108

**Published:** 2024-10-31

**Authors:** Kevin John

**Affiliations:** ^1^ University of Canberra, Research Institute for Sport and Exercise Bruce Australian Capital Territory Australia

**Keywords:** heat therapy, limb disuse, thermal garment

## Abstract

Partaking in regular exercise has vast psychological and physiological benefits. However, factors that promote sedentary lifestyle such as occupational obligations (desk‐based work) or underlying health comorbidities can limit adherence to exercise regimes. Considering the current trends in physical inactivity, development of alternate strategies to replicate or mimic the beneficial adaptations associated with regular exercise may become a highly sought after commodity. A relevant and current example of this is the enormous market demand for glucagon‐like peptide‐1 drugs for the management of obesity and type‐2 diabetes. The goal of this short review is to direct attention toward non‐pharmaceutical strategies and specifically focuses on the topical application of heat stress to passively improve health. The review highlights important heat‐induced adaptations and identifies scope for technological innovations that will allow delivery of heating interventions outside the confinement of laboratory settings.

## 1 INTRODUCTION

There is overwhelming evidence supporting the health sustaining effects of exercise and its medicinal potential, aiding in the treatment of various pathophysiological conditions (Pedersen & Saltin, 2015). However, a significant proportion of the world's population do not meet minimum physical activity recommendations, and unsurprisingly, inactivity is a causal factor for various non‐communicable diseases (Lee et al., 2012). The factors related to poor activity levels include but are not limited to age, biological sex, and socioeconomic status (Lee et al., 2012). Perhaps a more significant obstacle to an active lifestyle is the presence of any disability or cardiometabolic disease, which prohibits or limits an individual's ability/capacity to exercise.

**FIGURE 1:**
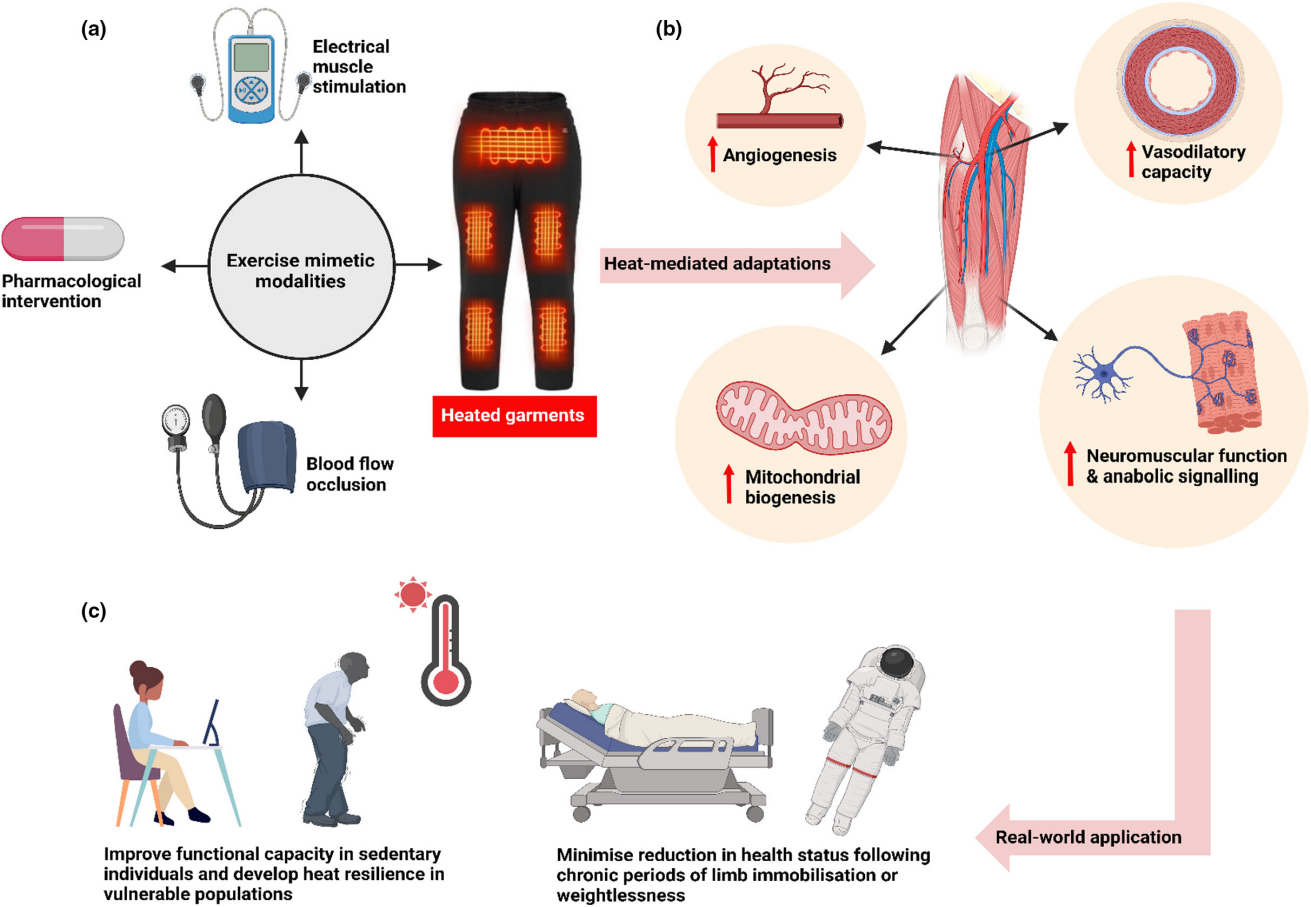
A graphical summary for the short review. Current examples of exercise mimetics (a) heat mediated improvements in specific physiological markers (b) and potential scenarios in which heating garments maybe beneficial for preserving health status (c). Created with BioRender.com.

Within this context, the development of exercise mimetics (Figure 1) could play an important role in the future of exercise physiology research. Traditionally, the term exercise mimetics has been reserved to the field of pharmacology; however, the current review defines it as any passive strategy (synthetic compound, invasive and non‐invasive procedures) that aims to artificially replicate the physiological stress imposed by exercise, completely or in‐part, for the purposes of inducing beneficial phenotypic adaptations in a sedentary human or animal. Some examples of non‐pharmaceutical exercise mimetics include intermittent blood‐flow restriction (artery occlusion) and electrical muscle stimulators that are based on the principle of mimicking the local ischemic stress and pressure overload associated with voluntary muscular contractions. This short review will focus on yet another strategy—the topical application of heat, referred to as heat therapy—which has been culturally practiced for centuries in parts of Asia and Europe.

## 2 ADAPTATIONS TO REPEATED THERMAL STRESS

The cardiovascular adjustments to peripheral (increase in local tissue temperature) or whole‐body hyperthermia have been well defined (Rowell, 1983). Briefly, these involve redistribution of blood flow from the central to the peripheral vasculature, facilitated by selective sympathetic vasodilatory and vasoconstrictive neural responses, along with elevations in cardiac output. Interestingly, alterations at the level of the heart (i.e., ventricular contractile mechanics) are not always necessary for increasing cardiac output, with recent evidence suggesting a pre‐dominance of local mechanisms (increase in red blood cell temperature and vasodilation of microvasculature) explaining the increase in hemodynamic redistribution during regional hyperthermic exposures (Watanabe et al., 2024). More importantly, the hyperthermia‐induced hyperemic response results in the generation of mechanical shear stress along the epithelial lining of the vasculature (Naylor et al., 2011), which in turn upregulates the expression of angiogenic factors such as nitric oxide, endothelial nitric oxide synthase, and vascular endothelial growth factors (Wragg et al., 2014). This also provides a valid mechanistic explanation for the improvements in conduit vessel function (Naylor et al., 2011) and angiogenesis (Hesketh et al., 2019) noted following chronic whole‐body or local heating.

The beneficial effects of heat stress are not only limited to the cardiovascular system. Recently, in an in vitro experiment (Ishii et al., 2023), it was shown that skeletal myofibrillar protein can be activated (i.e., sarcomere shortening) directly via heat stress (∼40°C) in a calcium‐independent manner, indicating a functional component of heat therapy. Additionally, the seminal experiments by Liu and Brooks (2012) on mice C2C12 myotubules provided initial evidence for the capacity of repeated heat stress to induce mitochondrial biogenesis. Similar to exercise, cellular studies have revealed the pathways of heat‐induced mitochondrial adaptation, which includes an acute oxidative disruption and generation of reactive oxygen species (ROS), followed by a signaling cascade involving the upregulation of AMP‐activated protein kinase, heat shock factor 1, heat shock proteins, and peroxisome proliferator‐activated receptor gamma coactivator 1‐alpha, that facilitate mitochondrial remodeling and biogenesis (Tamura & Hatta, 2017). Recent in vivo experiments have indeed confirmed this, with Hafen and colleagues (Hafen et al., 2018) reporting increased skeletal mitochondrial electron transport protein expression following only six consecutive short‐wave diathermy sessions. The well‐known saying from Louis Sullivan that “form follows function” also applies to heat therapy, with various laboratories (Rodrigues et al., 2020) reporting improvements in skeletal muscle contractile properties following repeated heat exposures, while data on hypertrophy remain inconclusive.

Taken together, the current evidence on heat application is favorable and may become a key interventional tool, especially in situations demanding health preservation. A relevant example of this would be periods of weightlessness as experienced in hospital settings (i.e., bed rest following injury or surgery) and during space flight missions. Though both scenarios impose a different physiological challenge, they induce similar detrimental effects on determinants of human function or exercise capacity. Morphologically, these include significant skeletal (Trappe et al., 2009) and cardiac muscle atrophy (Perhonen et al., 2001), along with reductions in vascular compliance due to intima thickening (van Duijnhoven et al., 2010). Interestingly, recent studies applying heat therapy during unilateral limb immobilization in humans (Hafen et al., 2019; Hyldahl et al., 2021) have reported preservation of mitochondrial oxidative capacity, along with significant reductions in the rate of skeletal muscle atrophy and decline in conduit vessel function. The mechanisms explaining the protective effect of heat application during chronic periods of limb unloading are yet to be completely revealed; however, early work in mice indicates a reduction in cellular oxidative stress (Selsby & Dodd, 2005). As such, the age‐old practice of topical heat application may become a prominent therapeutic tool to mimic the beneficial health outcomes of exercise.

## 3 FABRIC INTEGRATED WITH HEATING ELEMENTS

At present, the wide scale application of heating interventions is limited, given that the thermal stimulus is dependent on specialist equipment (short‐wave diathermy, steam circulating suits, heat chambers, saunas and temperature‐controlled baths) requiring trained personnel and, in some instances, may be less tolerable or logistically impractical, reducing treatment compliance. However, given the recent advances in textile technology, it has been made possible to integrate heating elements within clothing. To the best of the authors' knowledge, only one study till date has investigated the effects of a remote post‐exercise heating intervention using electrical garments on markers of health and performance in recreationally active adults (John et al., 2024). Quite surprisingly, it was shown that 20 limb heating sessions (across 4 weeks) in addition to routine endurance training impaired pulmonary oxygen kinetics compared to exercise only controls (John et al., 2024), highlighting the importance of carefully considering the thermal dose when designing heating interventions. Though this finding is unusual, it mimics the well‐known concept of training periodization, whereby an adaptive stimulus or stressor ceases to be beneficial if it is misapplied. Another challenge with the heated garments currently available on the market is the development of heat spots due to poor heat distribution. A potential solution for this could be the implementation of carbon (allotropes) nano‐conductors (Vives & Tour, 2009) within textile fabric, which would allow the user to experience a more well‐rounded stimulus, with the added advantage of controlling the intensity of heating. Furthermore, given the current trends in climate change, the development of bespoke heating garments may contribute to building heat resilience in “at risk” populations. Indeed, the field of thermal therapy is undergoing exciting developments and further studies are required to investigate the use of practical heating interventions in clinical settings and highlight the key physiological mechanisms that explain the beneficial health outcomes, as well as clearly outline the appropriate dose required to induce these heat related adaptions.

## AUTHOR CONTRIBUTIONS

The author listed is the sole author.

## FUNDING INFORMATION

No funding was involved in the preparation of the manuscript.

## CONFLICT OF INTEREST STATEMENT

The author has no conflict of interest to disclose.

## ETHICS STATEMENT

None.
